# Effect of Metabolic Dysfunction-Associated Steatotic Liver Disease (MASLD) on Left Ventricular Mechanics in Patients Without Overt Cardiac Disease: A Systematic Review and Meta-Analysis

**DOI:** 10.3390/jcm14082690

**Published:** 2025-04-15

**Authors:** Andrea Sonaglioni, Federica Cerini, Valeria Fagiani, Gian Luigi Nicolosi, Maria Grazia Rumi, Michele Lombardo, Paola Muti

**Affiliations:** 1 Division of Cardiology, IRCCS MultiMedica, 20123 Milan, Italy; michele.lombardo@multimedica.it; 2 Hepatology Unit, IRCCS MultiMedica, 20123 Milan, Italy; federica.cerini@unimi.it (F.C.); mariagrazia.rumi@unimi.it (M.G.R.); 3 Department of Clinical Sciences and Community Health, Dipartimento di Eccellenza 2023–2027, University of Milan, 20122 Milan, Italy; 4 Department of Emergency, Fondazione IRCSS Ca’ Granda, Ospedale Maggiore Policlinico, 20122 Milan, Italy; valeria.fagiani@unimi.it; 5 Division of Cardiology, Policlinico San Giorgio, 33170 Pordenone, Italy; gianluigi.nicolosi@gmail.com; 6 Department of Biomedical, Surgical and Dental Sciences, University of Milan, 20122 Milan, Italy; pmuti26@gmail.com; 7 IRCCS MultiMedica, 20138 Milan, Italy

**Keywords:** metabolic dysfunction-associated steatotic liver disease, MASLD, subclinical myocardial dysfunction, left ventricular mechanics, GLS

## Abstract

**Background:** Over the last two decades, a fair number of echocardiographic studies have investigated the influence of metabolic dysfunction-associated steatotic liver disease (MASLD) on myocardial strain and strain rate parameters assessed by speckle tracking echocardiography (STE) in individuals without overt heart disease, reporting not univocal results. We aimed at analyzing the main findings of these studies. **Methods:** All studies examining conventional echoDoppler parameters by transthoracic echocardiography (TTE) and left ventricular (LV) mechanics [LV-global longitudinal strain (GLS), LV-global strain rate in systole (GSRs), in early diastole (GSRe) and late diastole (GSRl)] by STE in MASLD patients without known heart disease vs. healthy individuals, were searched on PubMed, Embase and Scopus databases. The primary endpoint was to quantify the effect of MASLD on LV-GLS in individuals without overt cardiac disease. Continuous data [LV-GLS, LV-GLSRs, LV-GLSRe, LV-GLSRl and left ventricular ejection fraction (LVEF)] were pooled as the standardized mean difference (SMD) comparing MASLD cohorts with healthy controls. **Results:** A total of 11 studies were included, totaling 1348 MASLD patients and 6098 healthy controls. Overall, MASLD showed a medium effect on LV-GLS (SMD −0.6894; 95%CI −0.895, −0.472, *p* < 0.001) and LV-GLSRs (SMD −0.753; 95%CI −1.501, −0.006, *p* = 0.048), a large effect on LV-GLSRe (SMD −0.837; 95%CI −1.662, −0.012, *p* = 0.047) and a small and not statistically significant effect on LV-GLSRl (SMD −0.375; 95%CI −1.113, 0.363, *p* = 0.319) and LVEF (SMD −0.134; 95%CI −0.285, 0.017, *p* = 0.083). The overall I^2^ statistic was 86.4%, 89.4%, 90.9%, 89.6% and 72.5% for LV-GLS, LV-GLSRs, LV-GLSRe, LV-GLSRl and LVEF studies, respectively, indicating high between-study heterogeneity. Egger’s test for LV-GLS studies gave a *p* value of 0.11, 0.26, 0.40, 0.32 and 0.42 for LV-GLS, LV-GLSRs, LV-GLSRe, LV-GLSRl and LVEF studies, respectively, thus excluding publication bias. Meta-regression analysis excluded any correlation between potential confounders and LV-GLS in MASLD individuals (all *p* > 0.05). Sensitivity analysis confirmed the robustness of study results. **Conclusions:** MASLD has a medium effect on LV-GLS, independently of demographics, anthropometrics and the cardiovascular disease burden. STE analysis may allow early detection of subclinical LV systolic dysfunction in MASLD patients, potentially identifying those who may develop heart failure later in life.

## 1. Introduction

Non-alcoholic fatty liver disease (NAFLD), defined as hepatic steatosis not related to a secondary cause of hepatic fat accumulation [1], has been recently renamed as metabolic dysfunction-associated steatotic liver disease (MASLD). This new definition requires the presence of at least one cardiometabolic risk factor in an individual with documented steatosis and the absence of harmful alcohol intake [2]. It represents the most common form of chronic liver disease worldwide, with a global prevalence of 30% [3]. MASLD is more frequently detected in the clinical practice due to the spread of unhealthy eating habits and sedentary lifestyle [4], the rising obesity rate in rural areas [5] and the population aging [6].

Recent epidemiological studies have demonstrated that MASLD is independently associated with increased risk of cardiovascular disease [7] and new-onset heart failure (HF) [8] in healthy adults. Notably, MASLD patients have a higher risk of developing heart failure with preserved ejection fraction (HFpEF), also after adjustment for baseline clinical and demographic factors [9]. The larger the extent of liver fibrosis, the higher the risk of coronary artery disease, adverse cardiac remodeling and cardiac arrhythmias (especially atrial fibrillation), which may precede and promote the HFpEF occurrence [10].

This evidence highlights the importance of identifying an early marker of myocardial dysfunction in MASLD patients without known heart disease. Recent innovations in ultrasound techniques have led to the development of speckle tracking echocardiography (STE), a noninvasive imaging modality that allows evaluation of the myocardial deformation properties of the left ventricle in the longitudinal, circumferential and radial directions [11]. An early impairment in the left ventricular (LV) global longitudinal strain (GLS), that is, the main STE-derived index of myocardial contractility, in the presence of preserved left ventricular ejection fraction (LVEF) (≥55%), has been reported in various clinical settings [12,13,14]. Importantly, LV-GLS has also been correlated with the degree of myocardial fibrosis (MF) detected on cardiac magnetic resonance [15] or on endomyocardial biopsy [16,17]. Therefore, LV-GLS is actually considered a marker of MF and a sensitive tool for early identifying subclinical myocardial dysfunction.

The possible association between liver fibrosis and MF, noninvasively assessed by LV-GLS, might clarify the mechanisms linking MASLD and HFpEF, thus allowing the clinicians to adopt preventive strategies for HF occurrence.

Over the last two decades, a fair number of echocardiographic studies have investigated the link between MASLD and early cardiac remodeling by using conventional transthoracic echocardiography (TTE) implemented with two-dimensional (2D) STE analysis. These studies were primarily designed for assessing LV-GLS magnitude in MASLD patients without overt cardiac disease, compared to healthy controls without MASLD. However, they reported not univocal results. Accordingly, the present systematic review and meta-analysis aimed at analyzing the main findings of these studies and at exploring the main pathophysiological mechanisms underpinning the subclinical myocardial dysfunction in MASLD patients without known heart disease.

## 2. Materials and Methods

This systematic review and meta-analysis was conducted following the PRISMA guidelines [18] and was registered in INPLASY (registration number INPLASY202530037).

### 2.1. Search Strategy

Two independent reviewers (A.S. and M.L.) performed an accurate search of all studies examining traditional echoDoppler variables by TTE and LV mechanics by STE in MASLD patients without overt cardiac disease, through February 2025, using PubMed, Embase and Scopus databases. The following terms were included in the search strategy: “non-alcoholic fatty liver disease” OR “NAFLD” OR “metabolic dysfunction-associated steatotic liver disease” OR “MASLD” AND “echocardiography” OR “speckle tracking echocardiography” AND “cardiac function” OR “left ventricular mechanics” OR “left ventricular global longitudinal strain” OR “left ventricular strain” OR “LV-GLS”. There was no specific timeframe for the inclusion of echocardiographic studies. There was no language restriction.

### 2.2. Eligibility Criteria

All case-control studies evaluating both conventional echoDoppler parameters by TTE and LV mechanics by STE in MASLD patients without known heart disease vs. healthy individuals without MASLD, regardless of their age, were included in this systematic review and meta-analysis. Criteria of exclusion were the following: echocardiographic studies focused on patients affected by metabolic dysfunction-associated steatohepatitis (MASH), liver fibrosis and liver cirrhosis; echocardiographic studies conducted in MASLD patients without LV-GLS assessment by STE, echocardiographic studies performed in MASLD patients without controls, studies that measured myocardial strain parameters with nonechocardiographic imaging techniques and without concomitant 2D-STE analysis, and finally published documents different from clinical articles.

### 2.3. Study Selection and Data Extraction

Based on the aforementioned eligibility criteria, two reviewers (A.S. and M.L.) screened the records and independently collected the following information concerning both MASLD patients and healthy controls: (1) demographics (age and sex); (2) anthropometrics [body surface area (BSA), body mass index (BMI) and waist circumference (WC)]; (3) prevalence of the most relevant cardiovascular risk factors (hypertension, smoking, type 2 diabetes, dyslipidemia and obesity); (4) hemodynamics [heart rate, systolic blood pressure (SBP) and diastolic blood pressure (DBP)]; (5) blood tests comprehensive of serum levels of transaminases and gamma-glutamyl transferase (GGT), glycometabolic parameters, estimated glomerular filtration rate (eGFR) [19], lipid profile and C-reactive protein (CRP); (6) conventional TTE-derived echoDoppler indices of cardiac chambers cavity size and function; (7) LV-GLS and left ventricular global strain rate magnitude in systole (LV-GSRs), in early diastole (LV-GSRe) and in late diastole (LV-GSRl) and additional data on LV-global circumferential strain (GCS), LV-global radial strain (GRS) and/or left atrial reservoir strain (LASr); finally, the current medical treatment.

### 2.4. Risk-of-Bias Assessment

The National Institutes of Health (NIH) Quality Assessment of Case-Control Studies was used to assess the risk of bias (RoB) [20]. For each study, the quality rating was independently estimated as “good”, “fair”, or “poor” by two authors (A.S. and G.L.N.). The level of agreement between the two raters was quantified by the Cohen’s Kappa coefficient [21].

### 2.5. Statistical Analysis

Continuous data were expressed as the median (range interquartile), whereas categorical variables as percentages (%). The primary endpoint was to quantify the effect of MASLD on LV-GLS in individuals without overt cardiac disease. Continuous data (LV-GLS, LV-GLSRs, LV-GLSRe, LV-GLSRl and LVEF) were pooled as the standardized mean difference (SMD) comparing MASLD cohorts with healthy controls. The overall SMDs of LV-GLS, LV-GLSRs, LV-GLSRe, LV-GLSRl and LVEF were calculated using the random-effect model, due to the high statistical heterogeneity among the included studies. The I-squared statistic (I^2^) was used to quantify the proportion of total variation between studies. Begg’s funnel plots and Egger’s test were used to assess potential publication bias. Meta-regression analysis was performed to explore the relationship between LV-GLS and several potential confounders, such as age, male sex, BMI, SBP, fasting plasma glucose (FPG), total cholesterol, anti-hypertensive therapy and the ultrasound system used for STE analysis. Finally, a sensitivity analysis was performed to evaluate the impact of removing each of the studies on the overall SMD of LV-GLS. The 95% confidence intervals (CIs) were calculated and two-tailed *p* values less than 0.05 were considered to be statistically significant. Comprehensive Meta-Analysis version 3.0 (Biostat, Englewood, NJ, USA) was the software employed to perform the statistical analysis.

## 3. Results

### 3.1. Study Selection

**Figure 1:**
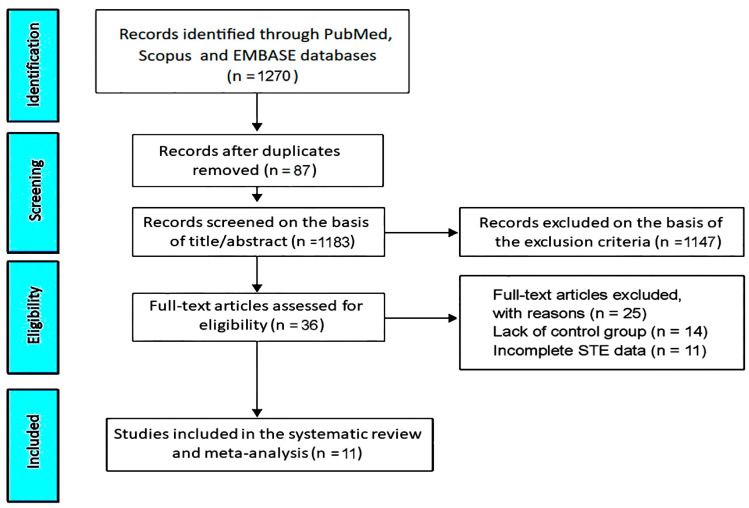
The PRISMA flow diagram used for identifying the included studies. PRISMA, Preferred Reporting Items for Systematic Reviews and Meta-analyses; STE, speckle tracking echocardiography.

The initial research performed in PubMed, Scopus and Embase databases gave rise to 1270 studies that evaluated cardiac function in MASLD patients. 87 studies (6.8%) were removed as duplicates. 1147 studies (90.3%) were excluded on the basis of the exclusion criteria. The remaining 36 studies (2.8%) were assessed for eligibility. Of these, 14 (1.1%) were excluded due to the lack of a control group of non-MASLD individuals and 11 (0.9%) due to incomplete STE data. Accordingly, 11 studies (0.9%) [22,23,24,25,26,27,28,29,30,31,32] were included in this systematic review and meta-analysis, totaling 1348 MASLD patients and 6098 healthy controls without MASLD (Figure 1).

### 3.2. Clinical Findings

The most salient features of the included studies are summarized in Table 1.

The included studies were published between 2012 and 2023. Three studies were performed in the USA, two in Italy and Turkey, one in Iran, Romania, Taiwan and Japan. The mean age of MASLD patients analyzed by the included studies was 47.7 years (range 15–68.6 years) and 63.9% (range 53.3–94.4%) of them were males. The great majority of studies (90.9%) had a prospective design, whereas only the study of Lai et al. [31] was retrospective. VanWagner et al. [27] and Chiu et al. [29] conducted multicentric population-based studies that analyzed participants from the CARDIA study and the Framingham Heart study, respectively; the remaining nine studies (81.8%) had a monocentric design. All the included studies evaluated MASLD patients with documented hepatic steatosis, at least one cardiometabolic risk factor and no evidence of steatohepatitis. The MASLD diagnosis was made on the basis of liver biopsy in three studies [24,25,28], of the fatty liver content assessed by liver ultrasonography in four studies [22,26,30,31], computed tomography (CT) in two studies [27,29] and magnetic resonance spectroscopy in one study [23], and of the fatty liver index (FLI) [33] in one study [32]. Concerning LV mechanics assessment, most studies (72.7%) evaluated LV myocardial strain and strain rate parameters by using a General Electric (GE) ultrasound software, two studies (18.2%) by a TomTec software and one study (9.1%) by a Toshiba ultrasound machine.

Table 2 reports all principal clinical, hemodynamic and biochemical parameters collected in MASLD patients and healthy controls by the included studies.

The most measured parameters were age, sex, BMI, blood pressure values, fasting glucose and serum levels of cholesterol, assessed by a percentage of studies ranging from 72.7% and 100% of total. Information concerning the prevalence of relevant cardiovascular risk factors, biochemical parameters and the current medical treatment was provided by a lower percentage of studies, ranging from one-third and two-thirds of total. Analysis of demographics and anthropometrics showed that MASLD patients were predominantly middle-aged males with a high prevalence of obesity. Compared to controls, MASLD individuals had a significantly higher prevalence of hypertension, type 2 diabetes and dyslipidemia, while the prevalence of smoking was similar in the two study groups. In regard to hemodynamics, both systolic and diastolic blood pressure values measured in MASLD patients by the included studies were significantly increased in comparison to those obtained in healthy controls, whereas heart rate was not statistically different in the two groups of individuals. On blood tests, liver enzymes, GGT and all main glycometabolic parameters were significantly increased in MASLD patients than controls; eGFR was significantly, although modestly, lower in MASLD individuals compared to controls; analysis of lipid profile revealed significantly increased serum levels of total cholesterol, low-density lipoprotein cholesterol and triglycerides and significantly reduced serum levels of high-density lipoprotein cholesterol in MASLD individuals vs. controls; finally, serum levels of CRP were significantly higher in MASLD patients than controls. Cardioprotective drugs were prescribed to a percentage of MASLD patients ranging from one-third and half of total. Among the antihypertensive drugs, angiotensin-converting-enzyme inhibitors (ACEIs) or angiotensin II receptor blockers, calcium channel blockers, beta blockers and diuretics were prescribed to 52.1%, 34.9%, 28.4% and 27.5% of MASLD individuals, respectively, whereas statins were used by 42.5% of MASLD patients. Antihypertensive drugs, diuretics and insulin were more frequently taken by MASLD than controls, whereas statins and oral hypoglycemic agents were administered to similar proportions of cases and controls.

### 3.3. Conventional Echocardiography and Deformation Imaging Findings

All conventional TTE parameters and STE indices measured in MASLD patients and healthy controls are listed in Table 3.

Among the traditional TTE parameters, those more commonly assessed were LVEF (100% of the studies), followed by left ventricular mass index (LVMi), E/A ratio and E/e’ ratio (81.8% of the studies) and relative wall thickness (RWT) (72.7% of the studies), whereas the remaining parameters were reported by a percentage of studies ranging from 36.4% and 63.6% of total. Overall, MASLD patients were diagnosed with normal left-sided cardiac chambers cavity sizes, mild LV concentric remodeling and normal LV systolic function, as assessed by LVEF (av. value 62.3%). Analysis of LV diastolic function showed impaired LV relaxation (av. E/A ratio = 0.96) with the E/e’ ratio in the “gray-zone” between 8 and 13 (av. E/e’ ratio = 8.4). Concerning LV myocardial deformation parameters, all the included studies measured LV-GLS, whereas LV-GSR, LV-GCS and LV-GRS were calculated in a percentage of studies ranging from 9.1% and 45.5% of total; differently from the other studies, the study of Lai Y.H. et al. [31] evaluated not only LV mechanics but also LASr and left atrial (LA) stiffness. All myocardial strain and strain rate parameters assessed in MASLD patients were slightly, but significantly, reduced in comparison to those obtained in non-MASLD individuals and to the accepted reference values [34,35]. However, LV-GLS magnitude was not statistically different in MASLD patients vs. controls in two studies [22,26].

**Figure 2:**
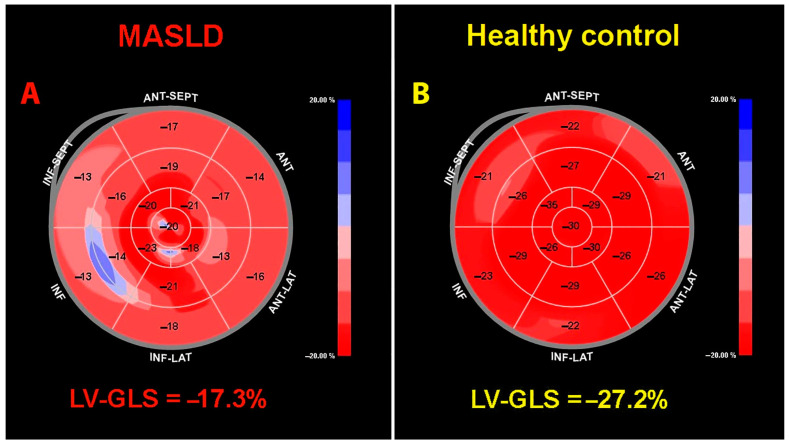
Representative examples of LV-GLS bull’s eye plot obtained by STE in an individual with MASLD (**A**) and in a healthy control (**B**). GLS, global longitudinal strain; LV, left ventricular; MASLD, metabolic dysfunction-associated steatotic liver disease; STE, speckle tracking echocardiography.

Figure 2 depicts two representative examples of LV-GLS bull’s eye plot obtained by STE in an individual with MASLD (A) and in a healthy control (B).

### 3.4. NIH Quality Rating

The NIH quality rating was estimated as good for seven studies and fair for four studies (Table 4).

The estimated Cohen’s Kappa coefficient k was 0.80, indicating a substantial agreement between the reviewers in the RoB assessment.

### 3.5. Effect of MASLD on LV-GLS

**Figure 3:**
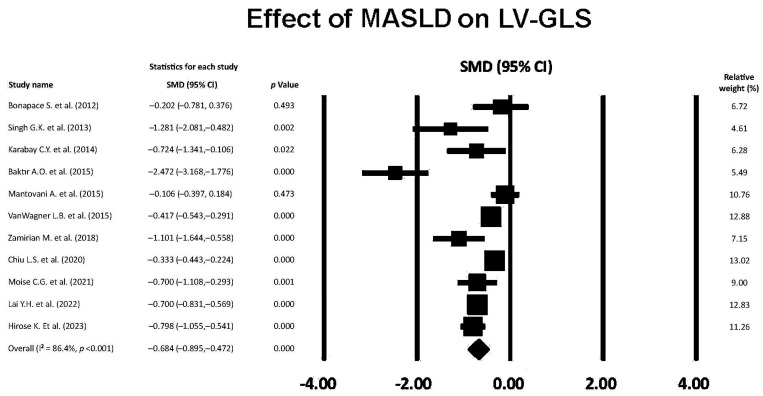
Forest plot showing the influence of MASLD on LV-GLS in the included studies [22,23,24,25,26,27,28,29,30,31,32]. GLS, global longitudinal strain; LV, left ventricular; MASLD, metabolic dysfunction-associated steatotic liver disease.

The forest plot showing the effect of MASLD on LV-GLS, assessed by the included studies, is illustrated in Figure 3.

**Figure 4:**
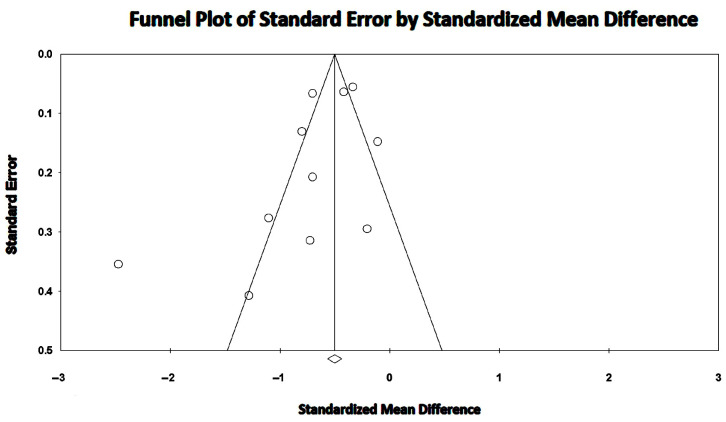
Begg’s funnel plot for the detection of publication bias in LV-GLS studies. GLS, global longitudinal strain; LV, left ventricular.

Overall SMD of LV-GLS was −0.6894 (95% CI −0.895, −0.472, *p* < 0.001), indicating a medium effect of MASLD on LV-GLS magnitude. The overall I^2^ statistic value was 86.4%, corresponding to high level of between-study heterogeneity. Egger’s test gave a *p* value equal to 0.11, thus excluding publication bias. Figure 4 shows the Begg’s funnel plot for LV-GLS studies.

Meta-regression analysis excluded any correlation between several moderators (age, male sex, BMI, SBP, FPG, total cholesterol, anti-hypertensive therapy and non-GE ultrasound system) and LV-GLS in MASLD individuals (all *p* > 0.05) (Table 5).

Sensitivity analysis confirmed the robustness of the study results. The omission of each study caused a mild variability in SMD, from −0.671 (95% CI −0.898, −0.444, *p* < 0.001) to −1.156 (95% CI −2.129, −0.183, *p* = 0.02).

### 3.6. Effect of MASLD on LV-GLSRs

**Figure 5:**
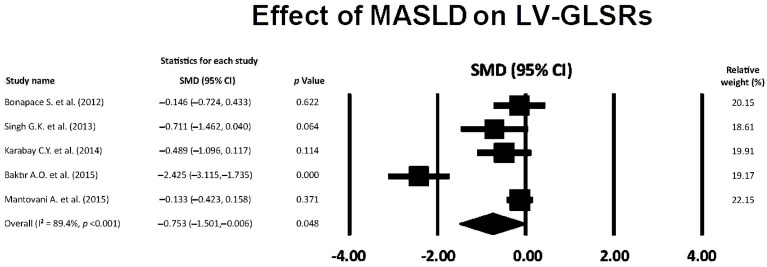
Forest plot showing the influence of MASLD on LV-GLSRs in the included studies [22,23,24,25,26]. GLSRs, global longitudinal strain rate in systole; LV, left ventricular; MASLD, metabolic dysfunction-associated steatotic liver disease.

The forest plot showing the effect of MASLD on LV-GLSRs, assessed by the included studies, is illustrated in Figure 5.

The overall SMD of LV-GLSRs was −0.753 (95% CI −1.501, −0.006, *p* = 0.048), indicating a medium effect of MASLD on LV-GLSRs magnitude. The overall I^2^ statistic value was 89.4%, compatible with substantial between-study heterogeneity. Egger’s test gave a *p* value equal to 0.26, thus excluding publication bias.

### 3.7. Effect of MASLD on LV-GLSRe

**Figure 6:**
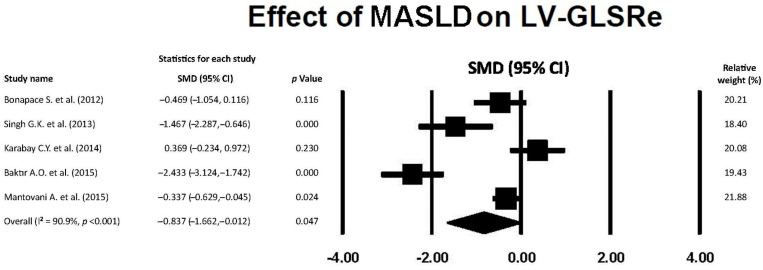
Forest plot showing the influence of MASLD on LV-GLSRe in the included studies [22,23,24,25,26]. GLSRe, global longitudinal strain rate in early diastole; LV, left ventricular; MASLD, metabolic dysfunction-associated steatotic liver disease.

The forest plot showing the effect of MASLD on LV-GLSRe, assessed by the included studies, is illustrated in Figure 6.

The overall SMD of LV-GLSRe was −0.837 (95% CI −1.662, −0.012, *p* = 0.047), indicating a large effect of MASLD on LV-GLSRe magnitude. The overall I^2^ statistic value was 90.9%, compatible with substantial between-study heterogeneity. Egger’s test gave a *p* value equal to 0.40, thus excluding publication bias.

### 3.8. Effect of MASLD on LV-GLSRl

**Figure 7:**
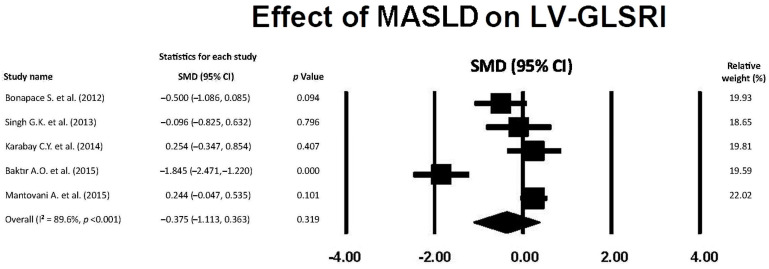
Forest plot showing the influence of MASLD on LV-GLSRl in the included studies [22,23,24,25,26]. GLSRl, global longitudinal strain rate in late diastole; LV, left ventricular; MASLD, metabolic dysfunction-associated steatotic liver disease.

The forest plot showing the effect of MASLD on LV-GLSRl, assessed by the included studies, is depicted in Figure 7.

The overall SMD of LV-GLSRl was −0.375 (95% CI −1.113, 0.363, *p* = 0.319), indicating a small and not statistically significant effect of MASLD on LV-GLSRl. The overall I^2^ statistic value was 89.6%, corresponding to a high level of between-study heterogeneity. Egger’s test gave a *p* value equal to 0.32, indicating no publication bias.

### 3.9. Effect of MASLD on LVEF

**Figure 8:**
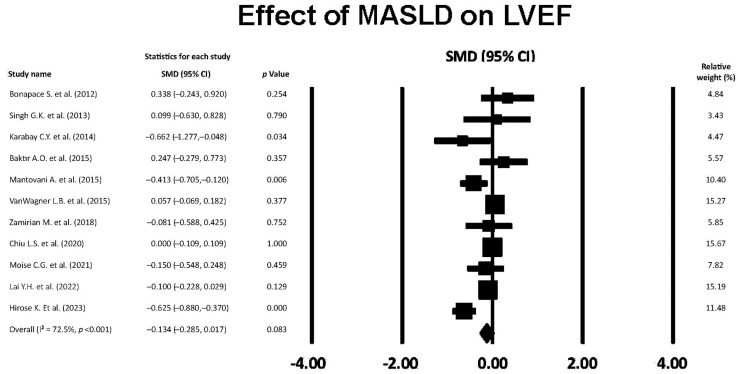
Forest plot showing the influence of MASLD on LVEF in the included studies [22,23,24,25,26,27,28,29,30,31,32]. LVEF, left ventricular ejection fraction; MASLD, metabolic dysfunction-associated steatotic liver disease.

The forest plot showing the effect of MASLD on LVEF, assessed by the included studies, is reported in Figure 8.

The overall SMD of LVEF was −0.134 (95% CI −0.285, 0.017, *p* = 0.083), indicating a small and not statistically significant effect of MASLD on LVEF. The overall I^2^ statistic value was 72.5%, indicating a high level of between-study heterogeneity. Egger’s test gave a *p* value equal to 0.42, indicating no publication bias.

## 4. Discussion

### 4.1. Main Findings

This systematic review and meta-analysis that analyzed 11 echocardiographic studies conducted between 2012 and 2023, including 1348 MASLD patients, revealed that these individuals (1) were predominantly middle-aged males with high prevalence of obesity and moderate prevalence of hypertension, type 2 diabetes and dyslipidemia; (2) were commonly found with hyperglycemia, hyperinsulinemia, insulin resistance (IR) and increased serum levels of CRP on laboratory tests; (3) had mild LV concentric remodeling with normal systolic function, grade I diastolic dysfunction and significantly higher LV filling pressures than controls on conventional TTE; (4) were diagnosed with significantly lower magnitude of all principal LV myocardial deformation indices compared to non-MASLD individuals on STE examination; (5) received cardioprotective drugs with a rate ranging from 27.5% to 52.1% of cases.

Meta-analysis results showed that MASLD had a medium effect on LV-GLS and LV-GLSRs, a large effect on LV-GLSRe and a small and not statistically significant effect on LV-GLSRl and LVEF. The effect of MASLD on LV-GLS magnitude was independent of several potential confounders, such as age, male sex, BMI, SBP, FPG, total cholesterol, anti-hypertensive therapy and non-GE ultrasound system. However, all these moderators, together with the different imaging techniques used for evaluating MASLD patients [conventional TTE, pulsed wave (PW)-tissue Doppler imaging (TDI) and STE] and the specific comorbidity burden of MASLD patients may account for the increased between-study heterogeneity detected for all the outcomes analyzed in this meta-analysis.

The Egger’s test excluded any publication bias for each outcome and the sensitivity analysis confirmed the independent effect of MASLD on LV-GLS.

The findings of this meta-analysis would suggest the occurrence of early cardiac remodeling and subclinical LV systolic dysfunction in MASLD individuals without overt heart disease. LV mechanics impairment detected in MASLD patients was characterized by the attenuation of LV systolic strain and LV strain rate in systole and early diastole, in the presence of preserved LVEF (≥55%).

### 4.2. LV Remodeling and LV Diastolic Dysfunction

Consistent with the literature data [36,37], the results of this systematic review and meta-analysis confirm that MASLD is associated with increased RWT and LVMi, indicating a LV remodeling in these patients. This remodeling may be the consequence of chronic volume and/or pressure overload, due to the concomitance of obesity and hypertension in most MASLD individuals.

Previous studies [36,37,38,39,40,41,42] have reported early LV diastolic dysfunction, as assessed by PW-TDI, in MASLD patients, both adults and children/adolescents, compared to individuals without steatosis. These studies demonstrated that MASLD was associated with LV diastolic dysfunction and increased LV filling pressures, independently from the main cardio-metabolic risk factors. The increase in the E/e’ ratio detected in MASLD patients has been considered as potential precursor of diastolic heart failure [22,27].

The preponderant effect of MASLD on LV-GLSRe, highlighted by our meta-analysis results, would indicate that the impairment in LV diastolic function might primarily involve the early diastolic filling, related to passive diastolic properties, whereas the active diastolic properties, ascribed to LA systole, might be subsequently altered.

### 4.3. Subclinical LV Systolic Dysfunction

**Figure 9:**
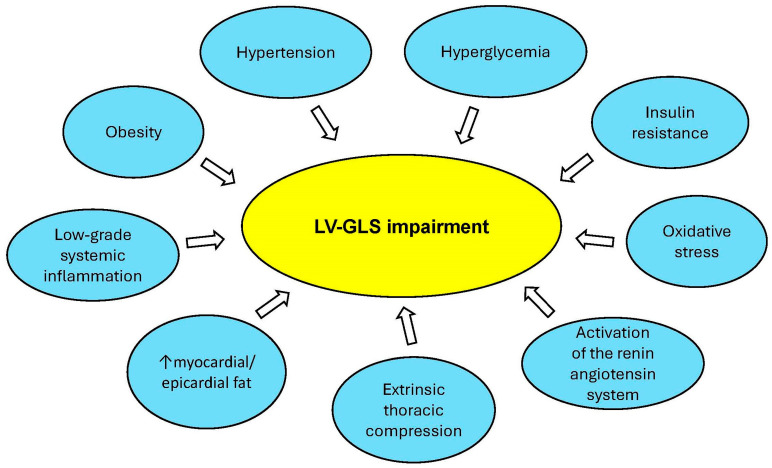
Pathophysiological mechanisms underpinning the association between MASLD and subclinical myocardial dysfunction. GLS, global longitudinal strain; LV, left ventricular; MASLD, metabolic dysfunction-associated steatotic liver disease.

Several pathophysiological mechanisms might explain the association between MASLD and subclinical myocardial dysfunction (Figure 9).

Literature data indicate that individuals with high BMI, such as those with overweight [43], obesity [44] and metabolic syndrome [45] are commonly diagnosed with subclinical impairment in LV-GLS. Consistent with this evidence, obesity might have an important role in determining the early deterioration in LV-GLS observed in MASLD patients.

The attenuation of myocardial strain parameters in MASLD patients has also been attributed to an increased fat accumulation in the myocardium/pericardium [46,47,48,49]; the higher the myocardial triglyceride content (myocardial steatosis), the greater the impairment of LV myocardial strain and strain rate indices.

Based on the lipotoxicity theory [47,48], MASLD, as a chronic inflammatory condition, may contribute to the overproduction of several systemic pathogenic mediators (such as CRP, interleukin-6, tumor necrosis factor-a, and other pro-inflammatory adipokines) which in turn may promote abnormal myocyte growth and fibrosis, and the activation of the sympathetic nervous system, finally leading to cardiac structural abnormalities, LV diastolic dysfunction and LV-GLS deterioration. Low-grade systemic inflammation in MASLD patients may impair the coronary microcirculation, thus causing the subclinical alteration in LV mechanics [50,51].

Among cardiovascular risk factors, arterial hypertension, that we have found in approximately half of the MASLD patients included in the meta-analysis, is another major determinant of LV-GLS reduction, as previously reported in non-MASLD patients [52].

As demonstrated in previous studies using STE analysis in asymptomatic, normotensive diabetic patients (both type 1 and type 2) with normal LVEF [53,54], chronic hyperglycemia may result in subclinical deterioration of myocardial contractility through an increase in oxidative stress [55,56].

As detected by the included studies, another important characteristic of MASLD is the insulin resistance (IR), occurring as result of increased inflammation and oxidative stress. IR is implicated in MASLD progression from steatosis to MASH [57] and may have a negative impact on LV systolic function in these individuals. It has been previously demonstrated that IR may negatively affect LV geometry and function, independently of the traditional risk factors [58,59,60,61].

LV systolic function may also be adversely affected by neurohormonal dysregulation, including the activation of the renin–angiotensin–aldosterone system and cardiac autonomic dysfunction [62,63].

All the above-mentioned factors would support the presence of an intrinsic myocardial systolic dysfunction in MASLD patients. In the different setting of healthy individuals with android obesity [64], our study group hypothesized that the LV-GLS impairment was primarily related to extrinsic thoracic compression on cardiac chambers, likely exerted by the combined action of abdominal and thoracic adiposity. This “mechanical theory” was supported by the evidence of a strong inverse correlation between LV-GLS and anthropometrics, such as the waist-to-hip ratio (WHR) and the modified Haller index (MHI) [65], detected in individuals with obesity, in absence of any intrinsic myocardial dysfunction.

### 4.4. Implications for Clinical Practice

Our findings confirm the incremental diagnostic value of STE analysis over conventional TTE for early detection of subclinical LV systolic dysfunction, as demonstrated in various non-MASLD study groups [66,67,68,69]. Indeed, LVEF, assessed by TTE, depends on good image quality for optimal visualization of the endocardial border and is strongly influenced by the operator’s experience for the correct identification of regional wall motion abnormalities [70]. For these technical reasons, although widely used in clinical practice, LVEF has poor sensitivity in detecting pre-clinical LV dysfunction [71].

Even if STE is actually available in most institutions, it is generally underutilized in clinical practice, mainly due to incomplete training and time constraints [72]. We firmly believe that STE should be considered for implementation in the echocardiographic assessment of MASLD patients, even if they are asymptomatic. STE analysis may allow the clinicians to early identify, among MASLD patients, those with subclinical impairment of LV deformation in the longitudinal direction, despite preserved LVEF (≥55%). In this regard, a strong inverse correlation between liver stiffness measurement (LSM), estimated by transient elastography, and STE-derived LV-GLS, has been recently demonstrated by our study group in MASLD patients without overt heart disease [73]. This finding would confirm that both liver fibrosis and myocardial fibrosis share common pathophysiological mechanisms. Accordingly, those MASLD patients with higher LSM at basal evaluation might benefit from early cardiological evaluation and closer hepatological follow-up.

The independent association between MASLD and subclinical LV systolic dysfunction, highlighted by the present meta-analysis, might explain the increased risk of HFpEF observed in MASLD patients during the course of their life. Detecting an early attenuation of LV-GLS magnitude in MASLD patients without overt heart disease might improve the prognostic risk stratification of these individuals, potentially identifying those who have an increased risk of developing HFpEF. Cardioprotective treatment should be promptly administered and adequately up-titrated to target doses in MASLD patients with increased burden of cardiovascular risk factors, early LV remodeling and subclinical deterioration of LV mechanics. Recent evidence indicates that physical activity [74], weight loss [75], ACEIs [76], statins [77] and sodium–glucose co-transporter 2 inhibitors [78] might improve hepatic steatosis and the underlying metabolic syndrome in MASLD patients. Future investigations are needed for evaluating if MASLD improvement may ameliorate LV mechanics, thus reducing the future occurrence of HFpEF.

### 4.5. Study Limitations

The most relevant limitations of the included studies were the small sample size of MASLD patients in most studies, the monocentric design for 81.8% of the studies and the lack of adjusted data in 63.6% of them. However, our meta-regression analysis excluded the possible influence of several confounders on LV-GLS magnitude in MASLD individuals.

Moreover, as detected by the I^2^ statistic value obtained for those studies assessing the various indicators of LV mechanics (LV-GLS, LV-GLSRs, LV-GLSRe, LV-GLSRl), a high between-study heterogeneity was observed. This finding was likely related to the inclusion of MASLD individuals from different countries, affected by various degrees of metabolic abnormalities, who underwent STE analysis by different vendors.

Additionally, the studies included in this meta-analysis used different methods to diagnose MASLD, such as liver ultrasonography, CT, magnetic resonance spectroscopy and the fatty liver index, whereas liver biopsy was performed in only 27.3% of the studies. Therefore, MASLD was not confirmed by liver biopsy in the majority of the included studies and other causes of liver disease could not be excluded. The clinical decision of not performing liver biopsy was primarily related to the detection of normal or only mildly increased liver enzymes in most MASLD patients. Considering the large number of MASLD patients encountered in clinical practice, this invasive procedure is actually recommended only for MASLD patients with increased risk of MASH or advanced fibrosis [79]. Conversely, hepatic ultrasonography is the most commonly used imaging modality for assessing the presence of hepatic steatosis, with good sensitivity and specificity especially in the presence of >30% fatty infiltration [80].

It is also important to consider that the reproducibility of STE analysis may be affected by the inter-vendor variability, operator’s experience, the quality of echocardiographic images, the frame rate setting, the loading conditions and, finally, extrinsic mechanical factors, particularly anterior chest wall deformity [81,82,83,84].

## 5. Conclusions

MASLD has a medium effect on LV-GLS, independently from demographics, anthropometrics and the cardiovascular disease burden.

STE analysis may allow early detection of subclinical LV systolic dysfunction in MASLD patients, potentially identifying those who may develop HFpEF later in life.

Future echocardiographic studies are warranted to establish if non-pharmacological and/or pharmacological treatments may reverse the impairment in LV mechanics in MASLD patients and/or prevent the future occurrence of HFpEF.

## Figures and Tables

**Table 1:** Clinical characteristics of the included studies and main echocardiographic findings detected in MASLD individuals vs. healthy controls. EDD, end-diastolic diameter; EDV, end-diastolic volume; ESD, end-systolic diameter; GCS, global circumferential strain; GE, General Electric; GLS, global longitudinal strain; GLSR, global longitudinal strain rate; GRS, global radial strain; LA, left atrial; LASr, left atrial reservoir strain; LAVi, left atrial volume index; LV, left ventricular; LVEF, left ventricular ejection fraction; LVMi, left ventricular mass index; MASLD, metabolic dysfunction-associated steatotic liver disease; RWT, relative wall thickness.

Study Name and Country	Number of Patients	Mean Age (yrs)	Males (%)	Study Design	Main Echocardiographic Findings in MASLD Patients vs. Healthy Controls
Bonapace, S. et al. (2012) [22], Italy	MASLD = 32 Controls = 18	MASLD = 64.8 Controls = 63	MASLD = 78.1 Controls = 72.2	Prospective	↔LVMi, LVEF, LAVi ↔E/A ratio, ↑E/e’ ratio ↔LV-GLS, GLSR in systole
Singh, G.K. et al. (2013) [23], USA	MASLD = 15 Controls = 14	MASLD = 15 Controls = 15	MASLD = 60 Controls = 53.3	Prospective	↑RWT, LVMi ↔LVEF ↓LV-GLS, GLSR in systole
Karabay, C.Y. et al. (2014) [24], Turkey	MASLD = 22 Controls = 21	MASLD = 42.8 Controls = 40.5	MASLD = 56.5 Controls = 57.1	Prospective	↑RWT, LVMi ↔LVEF, ↓E/A ratio, ↑E/e’ ratio ↓LV-GLS, GLSR in systole
Baktır, A.O. et al. (2015) [25], Turkey	MASLD = 28 Controls = 28	MASLD = 41.6 Controls = 41.2	MASLD = 57.1 Controls = 57.1	Prospective	↑RWT ↔LVEF, LAVi, E/A ratio, E/e’ ratio ↓LV-GLS, LV-GRS, ↔LV-GCS
Mantovani, A. et al. (2015) [26], Italy	MASLD = 158 Controls = 64	MASLD = 68.6 Controls = 66.9	MASLD = 63.9 Controls = 85.9	Prospective	↔LVMi, ↓LVEF, ↑LAVi ↔E/A ratio, ↑E/e’ ratio ↔LV-GLS, GLSR in systole
VanWagner, L.B. et al. (2015) [27], USA	MASLD = 271 Controls = 2442	MASLD = 50.5 Controls = 50.1	MASLD = 54.6 Controls = 39.7	Prospective	↑RWT, LVMi, LAVi ↔LVEF, ↓E/A ratio, ↑E/e’ ratio ↓LV-GLS, ↔LV-GCS
Zamirian, M. et al. (2018) [28], Iran	MASLD = 30 Controls = 30	MASLD = 38.4 Controls = 36.9	MASLD = 53.3 Controls = 50	Prospective	↓LV-EDD, LV-ESD ↔LVEF, E/A ratio, ↑E/e’ ratio ↓LV-GLS
Chiu, L.S. et al. (2020) [29], USA	MASLD = 384 Controls = 1972	MASLD = 53 Controls = 52	MASLD = 53.9 Controls = 47	Prospective	↑RWT, LVMi, ↓E/A ratio, ↑E/e’ ratio ↔LV-EDD, LV-ESD, LVEF ↓LV-GLS
Moise, C.G. et al. (2021) [30], Romania	MASLD = 35 Controls = 80	MASLD = 38 Controls = 29	MASLD = 57.1 Controls = 63.7	Prospective	↑LV-EDD, LV-ESD, RWT, LVMi ↔LVEF ↓LV-GLS, ↔LV-GCS
Lai, Y.H. et al. (2022) [31], Taiwan	MASLD = 302 Controls = 1019	MASLD = 56.4 Controls = 46.3	MASLD = 74.8 Controls = 50.7	Retrospective	↑RWT, LVMi, LAVi, LV-EDV ↔LVEF, ↓E/A ratio, ↑E/e’ ratio ↓LV-GLS, LASr, ↑LA stiffness
Hirose, K. et al. (2023) [32], Japan	MASLD = 71 Controls = 410	MASLD = 56 Controls = 57	MASLD = 94.4 Controls = 63.2	Prospective	↑LV-EDD, LV-ESD, RWT, LVMi ↓E/A ratio, ↔E/e’ ratio, LAVi ↓LV-GLS

**Table 2:** Clinical, hemodynamic and biochemical parameters collected in MASLD patients and healthy controls by the included studies. Data are expressed as the median and interquartile range. ACEi, angiotensin-converting-enzyme inhibitors; ALT, alanine transaminase; ARBs, angiotensin II receptor blockers; AST, aspartate transaminase; BB, beta blockers; BMI, body mass index; BSA, body surface area; CCB, calcium channel blockers; CRP, C-reactive protein; DBP, diastolic blood pressure; eGFR, estimated glomerular filtration rate; GGT, gamma-glutamyl transferase; HbA1C, glycosylated hemoglobin; HDL, high-density lipoprotein; HOMA-IR, homeostatic model assessment for insulin resistance; LDL, low-density lipoprotein; MASLD, metabolic dysfunction-associated steatotic liver disease; SBP, systolic blood pressure; WC, waist circumference.

	Number of Studies for Parameters Assessed (%)	Sample Size MASLD vs. Controls	MASLD	Controls	*p* Value
**Demographics**					
Age (yrs)	11 (100)	1348 vs. 6098	47.7 (15–68.6)	45.3 (15–66.9)	<0.05
Males (%)	11 (100)	1348 vs. 6098	63.9 (53.3–94.4)	58.2 (39.7–85.9)	<0.05
**Anthropometrics**					
BSA (m^2^)	3 (27.3)	690 vs. 4494	2.13 (2–2.3)	1.90 (1.8–2)	<0.05
BMI (Kg/m^2^)	11 (100)	1348 vs. 6098	30.3 (25.8–37)	25.5 (20–29.7)	<0.05
WC	5 (45.5)	698. 3910	101.2 (91.1–111.8)	89.3 (77.9–100)	<0.05
**Cardiovascular risk factors**					
Hypertension (%)	6 (54.5)	1218 vs. 5925	56.8 (30.8–81.6)	37.6 (8–73.4)	<0.05
Smoking (%)	7 (63.6)	968 vs. 4957	26.5 (10–46.5)	26.2 (10.7–50)	NS
Type 2 diabetes (%)	7 (63.6)	1253 vs. 5925	43.4 (0–100)	37.2 (1.7–100)	<0.05
Dyslipidemia (%)	3 (27.3)	644 vs. 3871	39.3 (11.9–56.7)	29.2 (4.2–50.2)	<0.05
Obesity (%)	2 (18.2)	293 vs. 2463	74.1 (68.2–80.1)	32.3 (23.8–40.9)	<0.05
**Hemodynamics (%)**					
Heart rate	4 (36.4)	240 vs. 176	76.6 (72.2–83)	77.1 (74.6–80)	NS
SBP (mmHg)	10 (90.9)	1318 vs. 6068	128.7 (120.7–143.9)	121.9 (109–139.7)	<0.05
DBP (mmHg)	10 (90.9)	1318 vs. 6068	79.4 (75–83.2)	74.2 (68–81)	<0.05
**Biochemical parameters**					
AST (U/L)	7 (63.6)	628 vs. 1574	31.5 (23–45.2)	23.8 (20–33)	<0.05
ALT (U/L)	7 (63.6)	628 vs. 1574	37.7 (24–66.1)	22.9 (15–33.4)	<0.05
GGT (U/L)	5 (45.5)	591 vs. 1539	49.1 (34–71)	26.7 (19–39.2)	<0.05
Fasting glucose (mg/dL)	8 (72.7)	1261 vs. 5967	117.8 (91–154.8)	106.8 (90–149.4)	<0.05
HbA1C (%)	5 (45.5)	834 vs. 3953	6.7 (6.1–7.5)	6.1 (5.5–7)	<0.05
Fasting insulin (U/L)	3 (27.3)	588 vs. 3475	22.6 (10.9–36)	8.3 (6.6–10.3)	<0.05
HOMA-IR	5 (45.5)	638 vs. 3524	5.3 (3.2–7.8)	1.8 (0.96–2.6)	<0.05
eGFR (mL/min/1.73 m^2^)	3 (27.3)	644 vs. 3871	85.8 (74–97.9)	87.6 (75–96)	<0.05
Total cholesterol (mg/dL)	9 (81.8)	1283 vs. 5988	194.7 (165–233.4)	183.9 (127–200.9)	<0.05
HDL-cholesterol (mg/dL)	9 (81.8)	1283 vs. 5988	46.9 (36–51.5)	53.2 (45–66)	<0.05
LDL-cholesterol (mg/dL)	9 (81.8)	1283 vs. 5988	120 (98.3–149)	109.9 (69–129.8)	<0.05
Triglycerides (mg/dL)	9 (81.8)	1283 vs. 5988	161.8 (120.5–217)	112.5 (64–167.3)	<0.05
CRP (mg/dL)	4 (36.4)	666 vs. 3892	2.1 (0.08–5.1)	1.3 (0.04–3.2)	<0.05
**Current medical treatment**					
ACE-i/ARBs (%)	4 (36.4)	645 vs. 2464	52.1 (21.1–77)	42.1 (14.4–68)	<0.05
CCB (%)	4 (36.4)	645 vs. 2464	34.9 (23.9–47)	24.4 (10.2–45)	<0.05
BB (%)	2 (18.2)	542 vs. 2036	28.4 (21.5–35.4)	13.3 (7.8–18.9)	<0.05
Diuretics (%)	2 (18.2)	190 vs. 82	27.5 (16–39)	18.8 (11–26.6)	<0.05
Statins (%)	4 (36.4)	645 vs. 2464	42.5 (25.3–74.1)	41.2 (17.3–79.9)	NS
Oral hypoglycemic agents (%)	3 (27.3)	261 vs. 492	49.4 (15.5–81.6)	45.1 (5.1–70.3)	NS
Insulin (%)	2 (18.2)	190 vs. 82	34.4 (33–35.8)	31.8 (23–40.6)	<0.05

**Table 3:** All conventional TTE parameters and STE indices measured in MASLD patients and healthy controls. Data are expressed as the median and interquartile range. EDD, end-diastolic diameter; EDV, end-diastolic volume; ESV, end-systolic volume; GCS, global circumferential strain; GLS, global longitudinal strain; GLSRe, global longitudinal strain rate in early diastole; GLSRl, global longitudinal strain rate in late diastole; GLSRs, global longitudinal strain rate in systole; GRS, global radial strain; IVS, interventricular septum; LASr, left atrial reservoir strain; LAVi, left atrial volume index; LV, left ventricular; LVEF, left ventricular ejection fraction; LVMi, left ventricular mass index; MASLD, metabolic dysfunction-associated steatotic liver disease; PW, posterior wall; RWT, relative wall thickness; STE, speckle tracking echocardiography; TTE, transthoracic echocardiography.

Echocardiographic Parameters	Number of Studies for Parameters Assessed (%)	Sample Size MASLD vs. Controls	MASLD	Controls	*p* Value
**TTE parameters**					
IVS thickness (mm)	5 (45.5)	417 vs. 1178	9.7 (8.6–11)	8.5 (8.2–8.8)	<0.05
LV-PW thickness (mm)	4 (36.4)	387 vs. 1148	10 (9.5–10.6)	8.4 (8.1–8.7)	<0.05
LV-EDD (mm)	6 (54.5)	570 vs. 2541	46.6 (41.9–49.1)	46.3 (44–49)	<0.05
RWT	8 (72.7)	1128 vs. 5986	0.42 (0.35–0.62)	0.39 (0.29–0.58)	<0.05
LVMi (g/m^2^)	9 (81.8)	1296 vs. 6040	87.7 (69.2–112.8)	78.9 (60–107.9)	<0.05
LV-EDV (mL)	7 (63.6)	1212 vs. 5625	100.3 (80.7–115.8)	97 (72.4–115.9)	<0.05
LV-ESV (mL)	6 (54.5)	910 vs. 4606	38.6 (22.4–46.6)	37.3 (24–42.9)	<0.05
LVEF (%)	11 (100)	1348 vs. 6098	62.3 (56.7–73.7)	62.9 (57.1–71.3)	<0.05
LAVi (mL/m^2^)	6 (54.5)	862 vs. 3981	26.5 (19.6–35.6)	24.2 (18.8–26.7)	<0.05
E/A ratio	9 (81.8)	1298 vs. 6004	0.96 (0.8–1.21)	1.13 (0.68–1.4)	<0.05
E/e’ ratio	9 (81.8)	1298 vs. 6004	8.4 (6.9–10)	6.7 (5.6–8.4)	<0.05
**STE indices**					
LV-GLS (%)	11 (100)	1348 vs. 6098	17.2 (7.7–19.9)	19.1 (14.8–23.7)	<0.05
LV-GLSRs (s^−1^)	5 (45.5)	255 vs. 145	1.0 (0.9–1.1)	1.2 (1–1.7)	<0.05
LV-GLSRe (s^−1^)	5 (45.5)	255 vs. 145	1.1 (0.8–1.3)	1.4 (0.9–2.3)	<0.05
LV-GLSRl (s^−1^)	5 (45.5)	255 vs. 145	0.9 0.5–1.2)	1.0 (0.5–1.5)	<0.05
LV-GCS (%)	3 (27.3)	334 vs. 2550	19.8 (15–23.6)	19.2 (15.4–23.3)	<0.05
LV-GRS (%)	1 (9.1)	28 vs. 28	41.1 (25.1–57.1)	57.2 (43.2–71.2)	<0.05
LASr (%)	1 (9.1)	302 vs. 1019	34 (26–42)	40.2 (32.8–47.6)	<0.05

**Table 4:** Quality Assessment of Case-Control Studies [22,23,24,25,26,27,28,29,30,31,32]. Q1–Q12 items are accessible from the following URL: https://www.nhlbi.nih.gov/health-topics/study-quality-assessment-tools (accessed on 28 February 2025).

NIH Quality Assessment Tool of Case-Control Studies Criteria Met													
Study Name	Q1	Q2	Q3	Q4	Q5	Q6	Q7	Q8	Q9	Q10	Q11	Q12	Quality
Bonapace, S. et al. [22]	Yes	Yes	No	Yes	Yes	Yes	NS	Yes	Yes	Yes	Yes	Yes	10 (Good)
Singh, G.K. et al. [23]	Yes	Yes	No	NS	Yes	Yes	NS	Yes	Yes	Yes	NS	No	7 (Fair)
Karabay, C.Y. et al. [24]	Yes	Yes	No	NS	Yes	Yes	NS	Yes	Yes	Yes	Yes	No	8 (Fair)
Baktır, A.O. et al. [25]	Yes	Yes	No	NS	Yes	Yes	NS	Yes	Yes	Yes	Yes	No	8 (Fair)
Mantovani, A. et al. [26]	Yes	Yes	No	NS	Yes	Yes	NS	Yes	Yes	Yes	Yes	Yes	9 (Good)
VanWagner, L.B. et al. [27]	Yes	Yes	No	Yes	Yes	Yes	Yes	Yes	Yes	Yes	NS	Yes	10 (Good)
Zamirian, M. et al. [28]	Yes	Yes	Yes	Yes	Yes	Yes	Yes	Yes	Yes	Yes	Yes	No	11 (Good)
Chiu, L.S. et al. [29]	Yes	Yes	No	Yes	Yes	Yes	Yes	Yes	Yes	Yes	Yes	Yes	11 (Good)
Moise, C.G. et al. [30]	Yes	Yes	No	NS	Yes	Yes	NS	Yes	Yes	Yes	NS	No	7 (Fair)
Lai, Y.H. et al. [31]	Yes	Yes	No	Yes	Yes	Yes	NS	Yes	Yes	Yes	Yes	No	9 (Good)
Hirose, K. et al. [32]	Yes	Yes	No	Yes	Yes	Yes	Yes	Yes	Yes	Yes	Yes	No	10 (Good)

**Table 5:** Meta-regression analysis performed to explore the relationship between LV-GLS and potential confounders. BMI, body mass index; FPG, fasting plasma glucose; GE, General Electric; GLS, global longitudinal strain; LV, left ventricular; SBP, systolic blood pressure.

Moderators	Coefficient	Standard Error	95%CI Lower	95%CI Upper	*p*-Value
Age	0.0116	0.0496	−0.0856	0.1087	0.81
Male sex	−0.0026	0.0162	−0.0344	0.0292	0.87
BMI	0.0688	0.1493	−0.2238	0.3615	0.65
SBP	0.0007	0.0104	−0.0197	0.0211	0.94
FPG	0.0387	0.0341	−0.0282	0.1056	0.26
Total cholesterol	−0.0093	0.0162	−0.0409	0.0224	0.57
Anti-hypertensive therapy	−0.0376	0.0374	−0.1109	0.0356	0.31
Ultrasound system: non-GE	−0.2826	1.1742	−2.5839	2.0188	0.81

## Data Availability

Data extracted from included studies will be publicly available on Zenodo (https://zenodo.org accessed on 28 February 2025).

## Abbreviations

2D, two-dimensional; ACEIs, angiotensin-converting-enzyme inhibitors; BMI, body mass index, BSA, body surface area; CIs, confidence intervals; CRP, C-reactive protein; CT, computed tomography; DBP, diastolic blood pressure; eGFR, estimated glomerular filtration rate; FPG, fasting plasma glucose; GCS, global circumferential strain; GE, General Electric; GGT, gamma-glutamyl transferase; GLS, global longitudinal strain; GRS, global radial strain; GSRe, global strain rate in early diastole; GSRl, global strain rate in late diastole; GSRs, global strain rate in systole; HF, heart failure; HFpEF, heart failure with preserved ejection fraction; IR, insulin resistance; LA, left atrial; LASr, left atrial reservoir strain; LSM, liver stiffness measurement; LV, left ventricular; LVEF, left ventricular ejection fraction; LVMi, left ventricular mass index; MHI, modified Haller index; MASH, metabolic dysfunction-associated steatohepatitis; MASLD, metabolic dysfunction-associated steatotic liver disease; MF, myocardial fibrosis; NAFLD, non-alcoholic fatty liver disease; NIH, National Institutes of Health; PW, pulsed wave; RoB, risk of bias; RWT, relative wall thickness; SBP, systolic blood pressure; SMD, standardized mean difference; STE, speckle tracking echocardiography; TDI, tissue Doppler imaging; TTE, transthoracic echocardiography; WC, waist circumference; WHR, waist-to-hip ratio.
